# Supplementary material for: cncFinder: A graph-attention-network-based interpretable learning model to identify bifunctional long non-coding RNAs
Source: Mol Ther Nucleic Acids. 2025 Dec 26;37(1):102812. doi: 10.1016/j.omtn.2025.102812 (PMC12830212; doi:10.1016/j.omtn.2025.102812)
Supplement: Document S1. Figure S1 and Tables S1–S3 [file mmc1.pdf]

**OMTN, Volume *37***

## Supplemental information

# cncFinder: A graph-attention-network-based interpretable learning model to identify bifunctional long non-coding RNAs

**Qiang Tang, Yang Yu, Min Shen, Lin Zhang, Xu Jia, and Juanjuan Kang**

**Supplemental information**

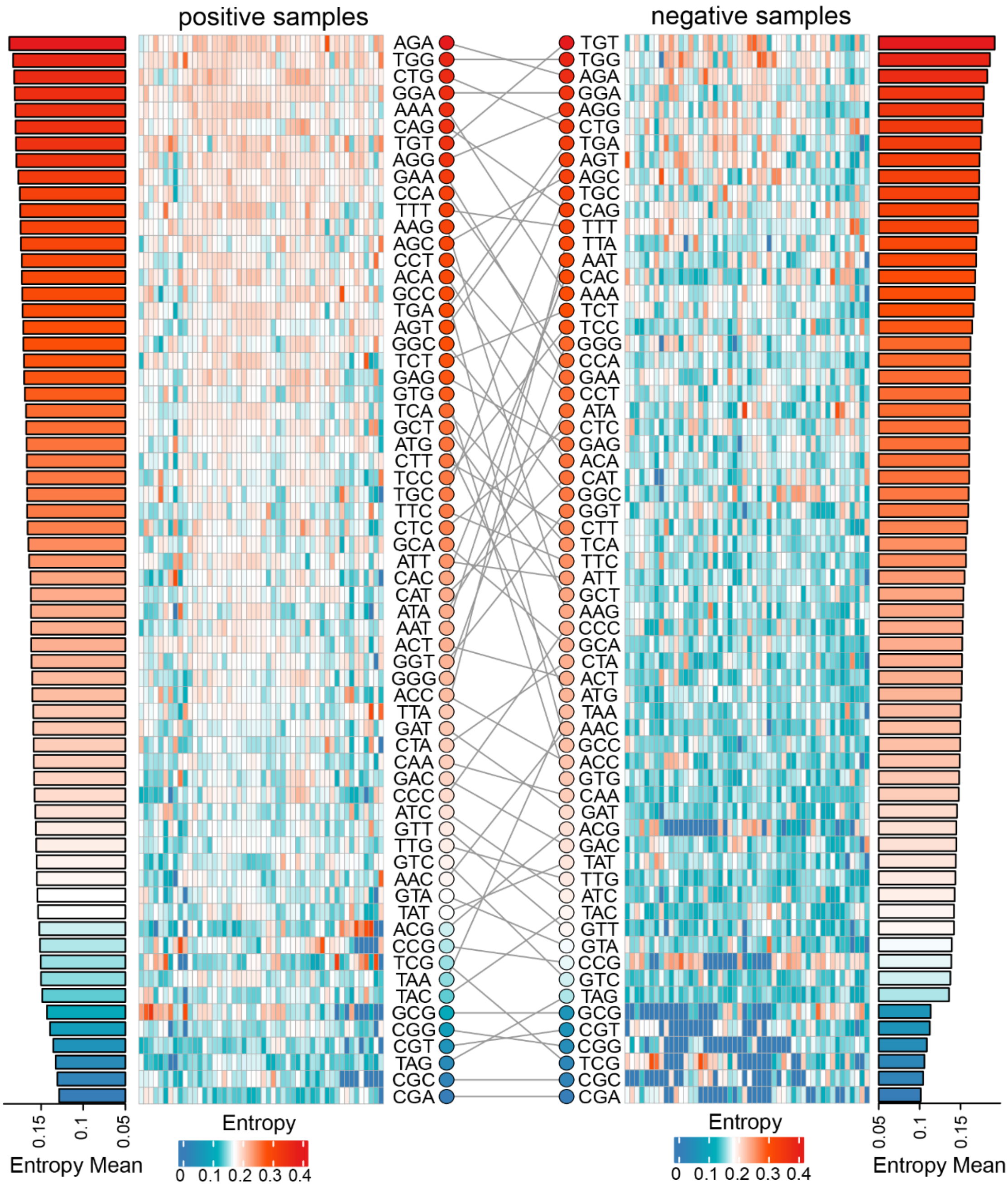


**Figure S1** Comparison of 3-mer entropy distributions between positive and negative samples. Left: Bar plot showing the average 3-mer attention entropy across 1,596 experimentally validated bifunctional lncRNAs. Heatmap displays the 3-mer entropy patterns for a randomly selected subset of 50 bifunctional lncRNAs. Right: Bar plot showing the average 3-mer entropy across 1,596 predicted non-bifunctional lncRNAs with the lowest prediction probabilities. Heatmap displays the 3-mer entropy for a randomly selected subset of 50 lncRNAs from this group.

**Table S1** Ablation Study.

| | Testing dataset | | | | | Cross-species dataset |
|---|---|---|---|---|---|---|
| Methods | SN | SP | ACC | MCC | AUC | ACC (No. correctly predicted) |
| Doc2Vec | 0.593 | 0.939 | 0.766 | 0.567 | 0.869 | 0.425(17) |
| fastText | 0.793 | 0.864 | 0.829 | 0.659 | 0.873 | 0.750(30) |
| TF-IDF | 0.885 | 0.563 | 0.724 | 0.473 | 0.854 | 0.825(33) |
| w/o GAT | 0.509 | 0.966 | 0.737 | 0.534 | 0.892 | 0.350(14) |
| GCN | 0.505 | 0.953 | 0.729 | 0.512 | 0.868 | 0.275(11) |
| cncFinder | 0.851 | 0.861 | 0.856 | 0.712 | 0.883 | 0.850(34) |

**Table S2** Comprehensive overview of dataset employed for cncFinder training and testing.

| Datasets | Species | Positive sequences | Negative sequences |
|---|---|---|---|
| Training | Human | 1,596 | 28,901 |
| Testing | Human | 295 | 295 |
| Cross species | Mouse | 34 | - |
| | Fruit fly | 6 | - |

**Table S3** The hyperparameter perturbations for cncFinder.

| Hyperparameters | Range |
|---|---|
| k-mer size | 3, 4, 5, 6 |
| Word2Vec embedding vector dimension | 64, 128, 256, 512 |
| number of hidden neurons | 64, 128, 256, 512 |
| number of attention heads | 3, 4, 5 |
