# Supplementary material for: cncFinder: A graph-attention-network-based interpretable learning model to identify bifunctional long non-coding RNAs
Source: Mol Ther Nucleic Acids. 2025 Dec 26;37(1):102812. doi: 10.1016/j.omtn.2025.102812 (PMC12830212; doi:10.1016/j.omtn.2025.102812)
Supplement: Document S2. Article plus supplemental information [file mmc2.pdf]

Molecular Therapy
Nucleic Acids
Original Article

cncFinder: A graph-attention-network-based interpretable learning model to identify bifunctional long non-coding RNAs

Qiang Tang,[1] Yang Yu,[2] Min Shen,[1] Lin Zhang,[3] Xu Jia,[1] and Juanjuan Kang[2,4]

[1]Key Laboratory of Non-coding RNA and Drug Discovery at Chengdu Medical College of Sichuan Province, School of Basic Medical Sciences, Chengdu Medical College, Chengdu 610500, China; [2]School of Intelligent Medicine, Innovative Institute of Chinese Medicine and Pharmacy, Chengdu University of Traditional Chinese Medicine, Chengdu 611137, China; [3]Department of Pharmacy, Shaoxing People's Hospital; Shaoxing Hospital, Zhejiang University School of Medicine, Shaoxing 312000, Zhejiang, China; [4]Affiliated Foshan Maternity & Child Healthcare Hospital, Southern Medical University (Foshan Maternity & Child Healthcare Hospital), Foshan 528000, China

Certain RNAs exhibit both protein-coding and regulatory non-coding functions, termed bifunctional RNAs or coding and non-coding RNAs. Long non-coding RNAs (lncRNAs), which play crucial roles in gene regulation and cellular processes, represent a major subset of bifunctional RNAs. Accurate identification of bifunctional lncRNAs is critical for advancing RNA biology and uncovering opportunities for biomarker discovery and therapeutic development. Here, we present cncFinder, a graph-attention-network-based model for predicting bifunctional lncRNAs. It transforms lncRNA sequences into k-mer graphs, encodes node features with Word2Vec, and employs graph attention network to capture higher-order sequence dependencies. On the testing dataset, cncFinder achieved superior performance, significantly outperforming state-of-the-art models. Its robustness and broad applicability were further confirmed through validation on cross-species datasets from mouse and fruit fly. Interpretability analysis revealed that cncFinder captured biologically meaningful motifs, including canonical start codons and Kozak-like elements. In a case study of LINC00961, cncFinder precisely detected an experimentally validated translation initiation motif, highlighting its biological relevance. To support broad accessibility, we developed a user-friendly web server. In summary, cncFinder advances predictive accuracy and interpretability, providing a powerful tool for systematic discovery of bifunctional lncRNAs and enabling new insights into RNA multifunctionality.

INTRODUCTION

RNAs are traditionally classified as either protein-coding (mRNAs) or non-coding (ncRNAs) according to their translational capacity.[1] However, accumulating evidence has revealed that some ncRNAs can also encode functional micropeptides, contributing to diverse biological processes.[2–4] These dual-function transcripts are referred to as bifunctional RNAs or coding and non-coding RNAs (cncRNAs).[2] Long non-coding RNAs (lncRNAs) represent a major subclass of ncRNAs, and a subset of them have been reported to exhibit bifunctionality, termed bifunctional lncRNAs.[5,6] For instance, LINC00961 encodes the polypeptide SPAR, which regulates mTORC1 activation and promotes muscle regeneration after acute injury.[7] Similarly, the putative lncRNA HOXB-AS3 encodes a peptide that suppresses colon cancer growth.[5] These findings highlight the functional complexity of lncRNAs and underscore the urgent need for systematic identification and characterization of bifunctional lncRNAs to deepen our understanding of RNA biology.

High-throughput experimental approaches such as ribosome sequencing (Ribo-seq)[8] and mass spectrometry (MS),[9] have been employed to characterize bifunctional lncRNAs. However, these techniques face substantial limitations. Ribo-seq assesses RNA translation efficiency by detecting ribosome-protected RNA fragments resistant to nuclease degradation.[8] However, ribosome occupancy alone is insufficient to establish coding potential, requiring additional computational refinement through tools such as RiboTaper[10] and ORFquant.[11] MS-based peptide identification also suffers from low peptide abundance, short half-lives, and technical sensitivity issues, especially for small peptides.[12] These challenges highlight the need for complementary computational methods capable of systematically and efficiently identify cncRNAs.

Machine learning, particularly deep learning, has been increasingly applied to functional RNA prediction.[13–16] The availability of experimentally validated cncRNAs has facilitated the development of curated resources such as cncRNAdb, which catalogs ∼2,600 cncRNAs, including more than 1,200 human bifunctional lncRNAs.[17]

Received 22 September 2025; accepted 22 December 2025;
https://doi.org/10.1016/j.omtn.2025.102812.

Correspondence: Xu Jia, Key Laboratory of Non-coding RNA and Drug Discovery at Chengdu Medical College of Sichuan Province, School of Basic Medical Sciences, Chengdu Medical College, Chengdu 610500, China.
E-mail: jiaxu@cmc.edu.cn
Correspondence: Juanjuan Kang, Innovative Institute of Chinese Medicine and Pharmacy, Chengdu University of Traditional Chinese Medicine, Chengdu 611137, China.
E-mail: kangjuanjuan@cdutcm.edu.cn

Published by Elsevier Inc. on behalf of The American Society of Gene and Cell Therapy.
This is an open access article under the CC BY license (http://creativecommons.org/licenses/by/4.0/).

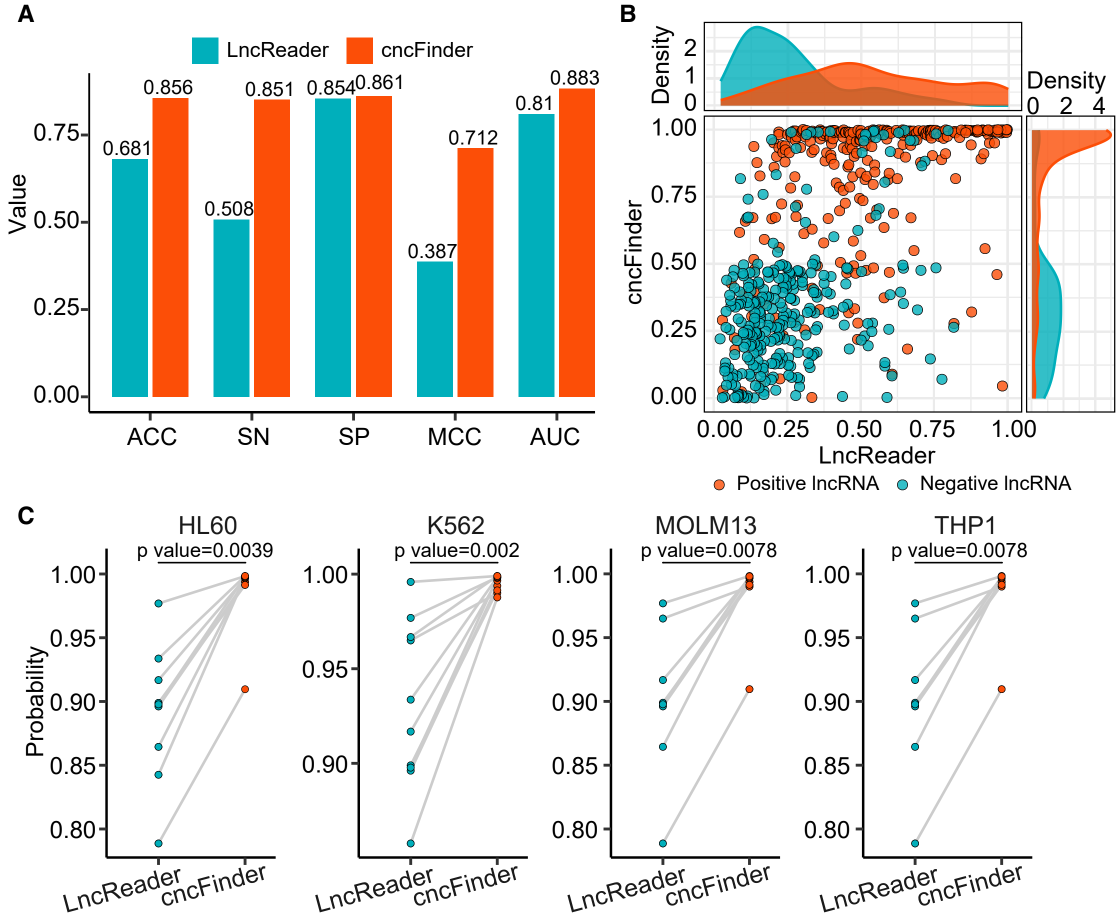

Figure 1. Performance evaluation on the testing dataset

(A) Comparison of evaluation metrics between cncFinder and LncReader. (B) Scatterplot of prediction probabilities assigned by cncFinder and LncReader. (C) Prediction probabilities across four cell lines. Statistical significance was assessed using the Wilcoxon rank-sum test. MOLM13 and THP1 shared an identical set of bifunctional lncRNAs.

Similarly, codLncScape compiles 353 experimentally confirmed coding lncRNAs to support bifunctionality research.[18] These databases provide valuable foundations for computational model development. Based on cncRNAdb, LncReader was proposed as the first deep learning framework utilizing multi-head self-attention to predict bifunctional lncRNAs.[19] Nonetheless, further advances are required to improve prediction accuracy and support systematic omics-based investigations of bifunctional lncRNAs.

To address these challenges, we developed cncFinder, a graph attention network (GAT)-based deep learning model for bifunctional lncRNA prediction. cncFinder transforms lncRNA sequences into k-mer-based directed graphs, encodes node features using Word2Vec, and leverages GAT to model complex inter-k-mer dependencies. A fully connected (FC) layer then classifies each transcript according to its bifunctional potential. cncFinder demonstrates superior accuracy and robustness across independent and cross-species datasets. To facilitate accessibility and support the scientific community, we developed a publicly available and user-friendly web server for cncFinder, freely accessible at http://i-health.info/cncFinder/.

RESULTS

Performance evaluation compared with LncReader

To evaluate the effectiveness of cncFinder, we compared its performance with LncReader on the testing dataset using a default classification threshold of 0.5. As presented in Figure 1A, cncFinder exhibited consistently strong performance, yielding an accuracy (ACC) of 0.856, sensitivity (SN) of 0.851, specificity (SP) of 0.861, Matthew's correlation coefficient (MCC) of 0.712, and the area under the receiver operating characteristic curve (AUC) of 0.883. In contrast, LncReader exhibited relatively lower values (ACC = 0.681, SN = 0.508, SP = 0.854, MCC = 0.387, and AUC = 0.810). Although the SP of both models was comparable, cncFinder markedly outperformed LncReader in terms of ACC, SN, and MCC, indicating a more balanced and robust classification performance.

The probability scatterplot in Figure 1B further illustrates these differences. Compared with LncReader, cncFinder consistently assigned higher prediction probabilities to positive lncRNAs and lower probabilities to negative lncRNAs, resulting in a more distinct separation between positive and negative samples.

Since the independent testing dataset of LncReader was derived from four cell lines (HL60, K562, MOLM13, and THP1), we further evaluated cncFinder on the same dataset to ensure a fair comparison. As shown in Figure 1C, cncFinder consistently assigned higher prediction probabilities to bifunctional lncRNAs than LncReader (Wilcoxon rank-sum test, $p < 0.01$).

Collectively, these results demonstrate that cncFinder achieved superior classification performance and generalization than LncReader.

Evaluation of cncFinder across different species

Because of the limited availability of bifunctional lncRNAs in non-human species, the current data are insufficient to construct species-specific prediction models. Nevertheless, cross-species predictive capability remains crucial for discovering novel bifunctional lncRNAs. To assess this, bifunctional lncRNAs from mouse and fruit fly were utilized to evaluate the cross-species predictive performance of cncFinder.

As illustrated in Figures 2A and 2B, cncFinder assigned significantly higher probabilities to cross-species datasets compared to LncReader, and the distributions of prediction probability differed markedly between the two models across species (Wilcoxon rank-sum test, $p < 0.05$). Moreover, Figure 2C shows that the probability density curves from cross-species predictions closely resembled those observed in the independent testing set. Compared with LncReader, cncFinder's prediction probabilities were more strongly skewed toward 1.0, with a higher average value (0.785 vs. 0.605), highlighting its superior discriminative power in cross-species scenarios. To further examine robustness under different classification thresholds, we plotted the ACC curves across a range of thresholds. As illustrated in Figure 2D, cncFinder consistently outperformed LncReader at all thresholds. At the default threshold of 0.5, cncFinder achieved a cross-species accuracy of 0.85, significantly higher than the 0.65 obtained by LncReader.

Together, these findings demonstrate that cncFinder exhibits superior accuracy and cross-species generalization, providing strong support for the identification of novel bifunctional lncRNAs.

Model effectiveness and stability analysis

To gain deeper insight into the internal mechanisms of cncFinder, we used Uniform Manifold Approximation and Projection (UMAP) to visualize the outputs of different model layers in two-dimensional space, thereby tracking the process of sample separation from a mixed to a separated state. As shown in Figure 3A, the initial features derived directly from input sequences displayed a random distribution, reflecting the raw and unprocessed nature of the data. After the sequences were transformed into graph structures, the samples began to exhibit an initial clustering trend (Figure 3B). This suggested that representing sequences as graphs provided an effective form of feature engineering that captured latent structural information. When the outputs were further processed by the GAT layer, the separation improved substantially, and the clusters of positive and negative samples became more compact and distinct with clearer boundaries (Figure 3C). Finally, as illustrated in Figure 3D, the outputs from the classification layer showed a well-defined separation between samples. These findings demonstrate that cncFinder effectively captures latent sequence features and enables precise sample discrimination, confirming the strength of its network architecture.

We then perturbed hyperparameters, including k-mer size, Word2Vec embedding dimension, number of hidden neurons, and number of attention heads. The baseline parameters were set to k-mer = 3, Word2Vec dimension = 64, hidden neurons = 64, and attention heads = 3. As shown in Figures 3E–3H, changes in the Word2Vec dimension and attention heads had little impact on performance, with all metrics remaining stable. Although modifications in k-mer size and hidden neuron number produced measurable changes in ACC, SN, SP, and MCC, the AUC demonstrated remarkable stability and consistently remained at a high level across configurations. Among the tested settings, the baseline combination achieved the best overall performance, confirming it as the optimal parameter configuration. Together, these findings indicate that cncFinder's effectiveness arises from its architectural design rather than reliance on specific hyperparameter choices.

Ablation study

To evaluate the contribution of different components in our framework, we conducted an ablation study. For node embedding, we replaced Word2Vec with alternative methods, including Doc2Vec, fastText, and TF-IDF. For graph learning, we compared the original GAT layer with alternative architectures, such as GCN, as well as a variant of cncFinder without the GAT layer (w/o GAT) (Figure 4; Table S1).

On the testing dataset, cncFinder achieved the best overall performance across all metrics. In contrast, traditional text-based embeddings, such as Doc2Vec and TF-IDF, exhibited markedly imbalanced sensitivity and specificity, indicating biased prediction tendency toward either positive or negative samples. By comparison, fastText achieved a more balanced trade-off between sensitivity and specificity (SN = 0.793, SP = 0.864), yet its overall performance remained inferior to that of cncFinder.

When the GAT layer was removed (w/o GAT), performance decreased substantially (ACC = 0.737, MCC = 0.534), underscoring the critical role of the GAT layer in capturing k-mer dependencies. Similarly, replacing GAT with GCN further reduced performance (ACC = 0.729, MCC = 0.512), suggesting that attention-based layer is more effective than convolutional layer for modeling bifunctional lncRNAs.

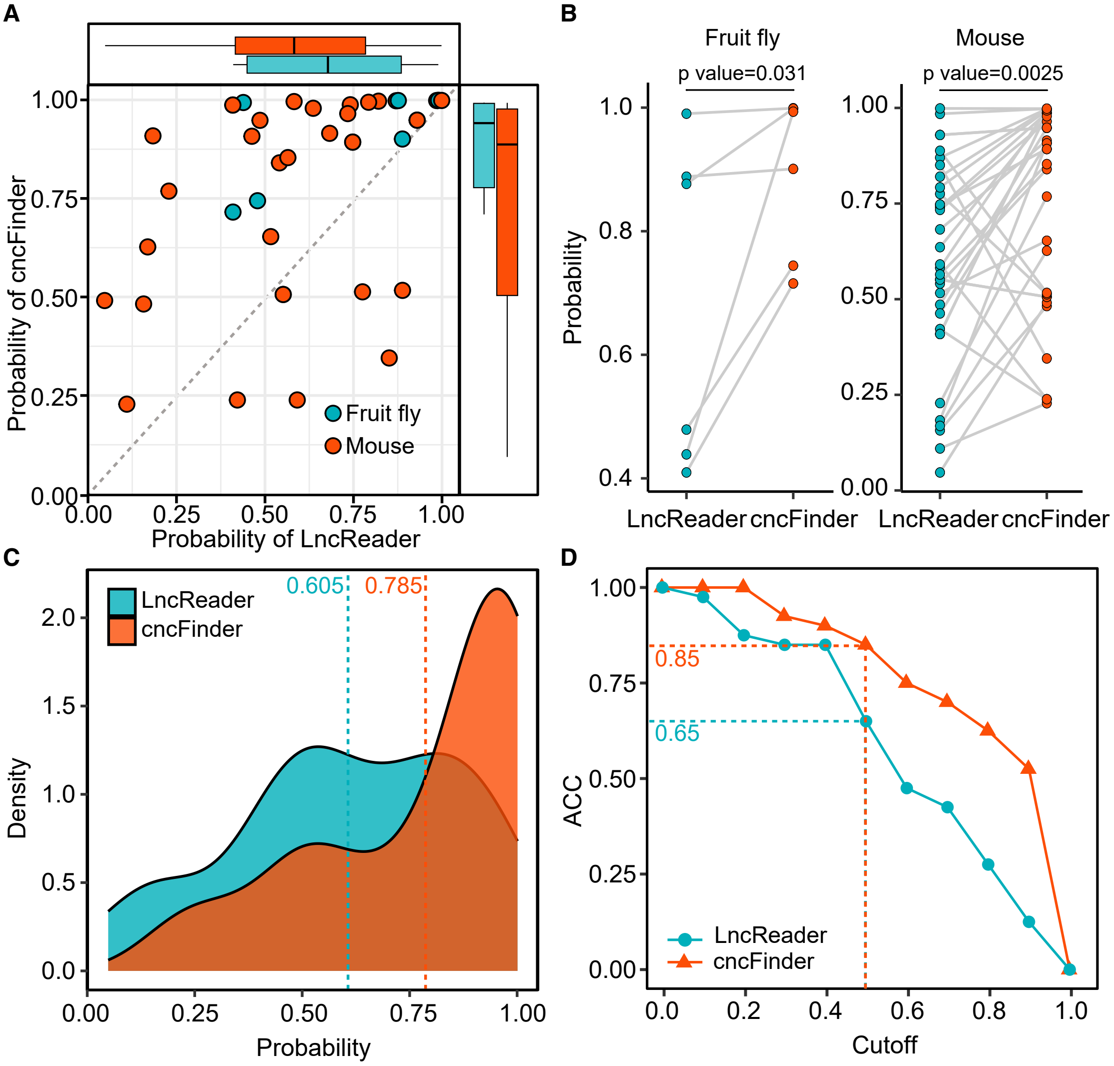

Figure 2. Cross-species prediction performance of cncFinder and LncReader

(A) Scatterplot of prediction probabilities for bifunctional lncRNAs from different species. (B) Comparison of prediction probabilities for different species; statistical significance was assessed using the Wilcoxon rank-sum test. (C) Probability density curve of predicted probabilities. (D) Accuracy comparison across varying classification thresholds.

On the cross-species dataset, cncFinder again outperformed all alternative methods, achieving the highest accuracy of 0.850. In comparison, TF-IDF and fastText obtained moderate performance (0.825 and 0.750, respectively), whereas Doc2Vec, GCN, and w/o GAT performed poorly. These results highlight that both the feature embedding strategy and the GAT layers are indispensable for cncFinder, and their integration confers strong predictive power and robust cross-species applicability.

Performance comparison with coding potential predictors

In addition to comparing cncFinder with LncReader, which was specifically designed for the identification of bifunctional lncRNAs, we also benchmarked its performance against two widely used coding potential predictors, CPC2[20] and PLEK.[21] Although CPC2 and PLEK were originally developed for distinguishing coding from non-coding transcripts rather than identifying bifunctional lncRNAs, they remain relevant for comparison because bifunctional lncRNAs inherently possess both coding and non-coding characteristics.

As shown in Table 1, both CPC2 and PLEK exhibited high specificity on the testing dataset (0.946 and 0.956, respectively), but achieved extremely low sensitivity (0.271 and 0.186), reflecting a strong bias to misclassify bifunctional lncRNAs as non-bifunctional. On the cross-species dataset, the ACCs of CPC2 and PLEK were only 0.225 and 0.300, respectively. In contrast, cncFinder consistently

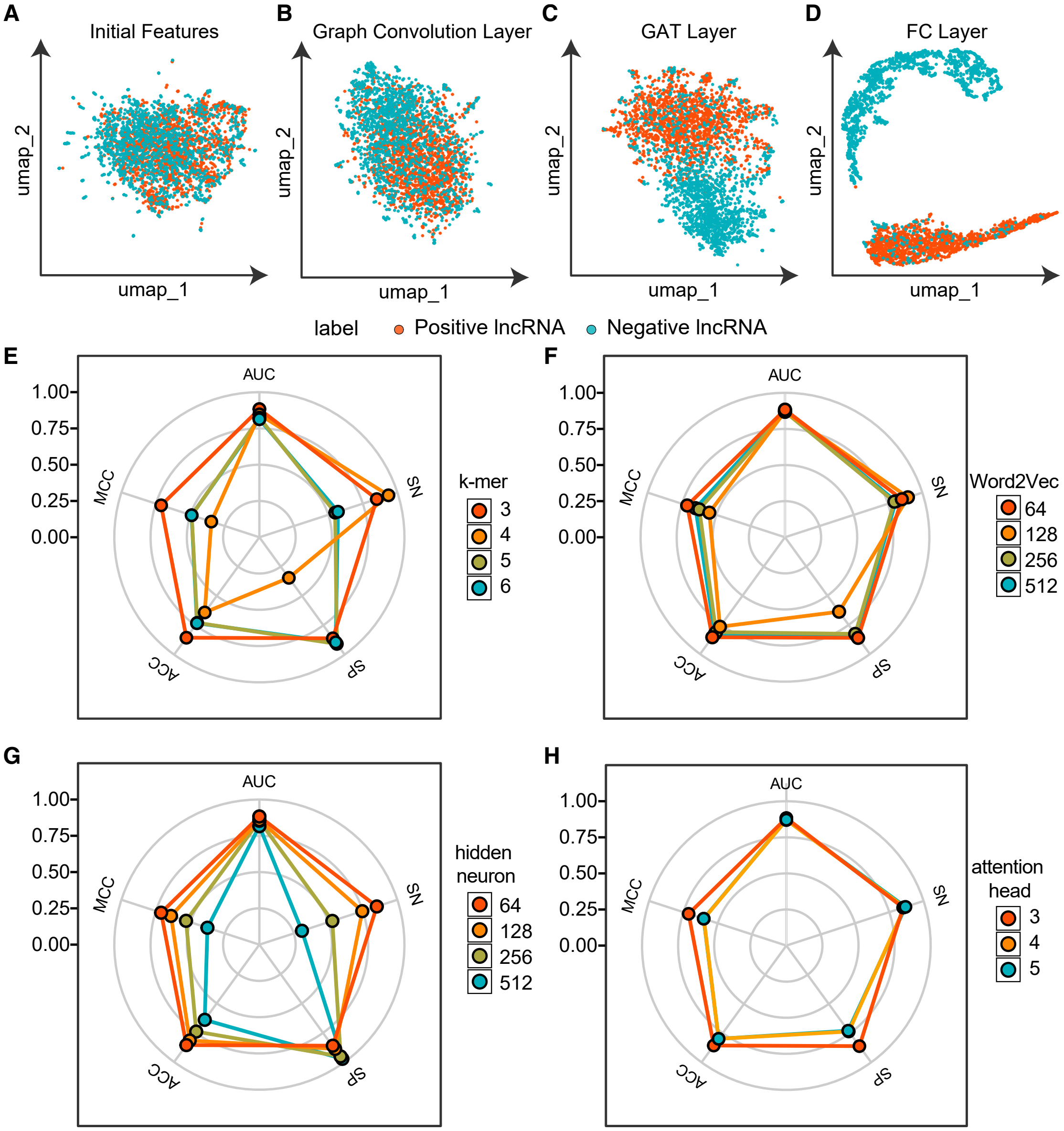

Figure 3. UMAP visualization and hyperparameter perturbation analysis of cncFinder

(A–D) UMAP visualization of features extracted from various layers of cncFinder. (A) Initial features, (B) graph output features before the GAT layer, (C) output from the GAT layer, and (D) output from the FC layer. (E–H) Model performance under different hyperparameter settings evaluated on the testing dataset. The evaluated hyperparameters include (E) k-mer size, (F) Word2Vec embedding vector dimension, (G) number of hidden neurons, and (H) number of attention heads.

outperformed both methods across all evaluations. These results underscore that identifying bifunctional lncRNAs is substantially more challenging than conventional coding potential prediction and further highlight the superior accuracy and robustness of cncFinder in addressing this task.

Interpretability analysis of cncFinder

To gain mechanistic insights into how cncFinder discriminates bifunctional lncRNAs, we computed the attention entropy of each 3-mer node based on edge weights from the GAT layer. We first examined the relationship between 3-mer entropy and occurrence frequency. A strong positive correlation was observed in both positive and negative samples (Figures 5A and 5B), indicating that frequently occurring 3-mers tended to exhibit more heterogeneous attention distributions. However, further analysis revealed qualitative differences in the correlation structures between entropy and frequency (Figure 5C). Specifically, the frequency correlation matrix displayed a clear modular architecture, which was divided into two

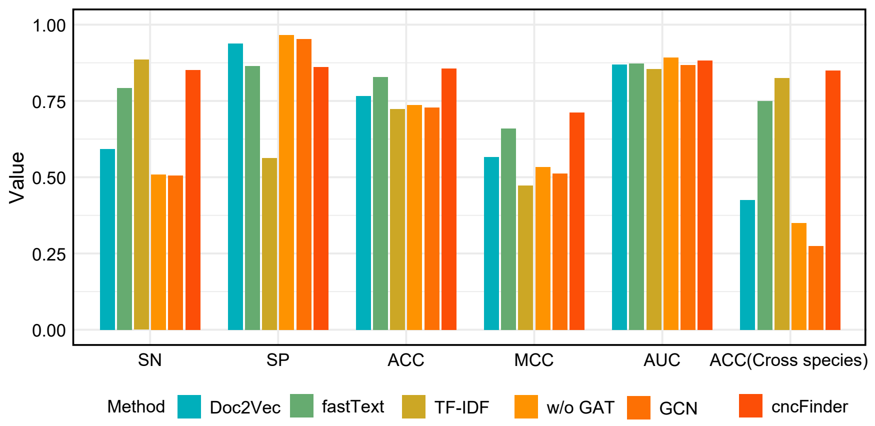

Figure 4. Performance evaluation of cncFinder and ablation variants on the testing dataset and the cross-species dataset

major clusters: one enriched for GC-rich 3-mers (e.g., CGG, GGC, and GCG) and another dominated by AT-rich 3-mers (e.g., TTA, AAA, and ATT). This pattern reflects compositional preferences and redundancy in 3-mer usage. In contrast, the entropy-based correlation network appears more diffuse and lacked obvious modularity, suggesting that cncFinder's attention mechanism captures higher-order contextual dependencies that extend beyond local composition. These results indicate that although frequency contributes to entropy, cncFinder encodes distinct and non-redundant representations of 3-mers shaped by their positional and contextual relevance within the sequences.

We next compared the distributions of 3-mer entropy between positive and negative samples and identified a statistically significant difference (Figure S1; Wilcoxon rank-sum test, $p = 2.45 \times 10^{-3}$). This finding suggests that cncFinder employs class-specific attention strategies. To further explore this distinction, we ranked 3-mers by their mean entropy within each class and identified those exhibiting the largest rank-order shifts between positive and negative samples (Figure 5D). In positive samples, high-ranking 3-mers such as ATG, GTG, GCC, and AAG were markedly enriched. These motifs are frequently associated with translation initiation, including canonical and non-canonical start codons (e.g., ATG and GTG)[22] as well as Kozak consensus elements (e.g., GCC).[23] Conversely, negative samples exhibited elevated entropy for 3-mers such as TAA, GGG, TTA, AAT, CAC, and TGC. These 3-mers typically lack canonical coding signals. Notably, termination codons such as TAA are often enriched in non-coding regions and have been recognized as important sequence features of lncRNAs.[24] These patterns suggest that cncFinder effectively distinguishes functionally relevant, coding-associated motifs, thereby enabling more accurate identification of bifunctional lncRNAs.

To further validate whether these attention-derived patterns localize meaningful biological features, we conducted a case study on LINC00961, a bifunctional lncRNA that encodes the micropeptide SPAR, which regulates mTORC1 signaling and muscle regeneration.[7] Within LINC00961, we observed a strong correlation between entropy and frequency, consistent with the global trends observed across the dataset (Figure 6A). High-entropy 3-mers included CAG and GCC, suggesting that these elements participate in diverse contextual interactions within the transcript. We then constructed a 3-mer interaction network based on attention weights (Figure 6B) and applied the edge percolated component (EPC) method to extract topologically central elements. Notably, ATG emerged as the highest-scoring 3-mer (Figure 6C), consistent with its role as the canonical translation initiation codon. Intriguingly, we identified a sequence motif "GCCATG" that conforms to the Kozak consensus sequence and is located at the second translation initiation site within the experimentally validated ORF of LINC00961 (Figure 6D). Moreover, a dataset-wide analysis showed that the "GCCATG" motif occurs significantly more frequently in bifunctional lncRNAs than in non-bifunctional lncRNAs (chi-squared test, $p = 2.62 \times 10^{-6}$). These findings suggest that the 3-mer network constructed by cncFinder can reveal biologically meaningful motifs, such as translation initiation sites, highlighting its potential to uncover functional sequence features and guide the discrimination of bifunctional lncRNAs.

Web server implementation

To enhance the accessibility of cncFinder, we developed a user-friendly web server, which is freely accessible at http://i-health.info/cncFinder/. Users can paste or upload RNA sequences of interest into the input box to obtain predictions of their bifunctional potential. The results page also provides detailed analysis of 3-mer frequency and entropy, along with the constructed attention network graph, facilitating deeper exploration of sequence features. This server aims to bridge the gap between computational model development and experimental research applications.

DISCUSSION

Bifunctional lncRNAs represent a unique class of transcripts capable of performing both regulatory and protein-coding functions. These dual roles position them as key players in diverse cellular processes, including gene regulation, signaling transduction, and stress responses. Therefore, accurate identification of bifunctional lncRNAs is fundamental for decoding RNA function and provides novel opportunities for therapeutic discovery.

In this study, we present cncFinder, a GAT-based deep learning framework for predicting bifunctional lncRNAs. Benchmarking on both testing and cross-species datasets showed that cncFinder consistently outperformed existing methods across all evaluation metrics. Moreover, interpretability analysis revealed that cncFinder

Table 1. Comparative evaluation with CPC2 and PLEK

	Testing dataset					Cross-species dataset
Methods	SN	SP	ACC	MCC	AUC	ACC (no. correctly predicted)
CPC2	0.271	0.946	0.609	0.294	0.746	0.225 (9)
PLEK	0.186	0.956	0.571	0.223	–	0.300 (12)
cncFinder	0.851	0.861	0.856	0.712	0.883	0.850 (34)

identified biologically meaningful sequence motifs associated with bifunctionality, such as translation initiation signals.

Beyond predictive accuracy, cncFinder broadens the scope of functional genomics research. It can be applied to large-scale, disease-related transcriptomic datasets, such as those involving tumorigenesis[25] and cancer therapy resistance,[26] to systematically identify and prioritize candidate bifunctional lncRNAs for further experimental validation. Such applications may facilitate the discovery of bifunctional lncRNAs as novel biomarkers or therapeutic targets, thereby offering new perspectives for disease diagnosis and treatment.

Nevertheless, several limitations warrant consideration. First, the current availability of experimentally validated bifunctional lncRNAs remains limited, particularly in non-human species, which constrains model training and benchmarking. Second, cncFinder currently relies solely on sequence information and does not incorporate structural or multi-omics features that may better capture the dual nature of bifunctional lncRNAs. Third, although cross-species evaluation indicates good generalizability, species-specific sequence biases and evolutionary divergence remain challenges for transferring prediction ability across distant organisms.

These limitations also highlight promising future directions. Integrating additional biological modalities, such as RNA secondary structure, proteomic evidence of translation, and epigenomic context, may further enhance prediction performance and mechanistic interpretability. Moreover, transfer learning frameworks could help mitigate data scarcity and improve cross-species generalization. As more experimentally verified bifunctional lncRNAs become available, large-scale training updates and benchmarking will further refine cncFinder.

In summary, cncFinder provides a robust, accurate, and biologically interpretable computational tool for predicting bifunctional lncRNAs. As a valuable resource, it has the potential to accelerate the discovery and functional characterization of cncRNAs, contributing to a deeper understanding of the complexity and multifunctionality of the RNA world. Looking ahead, integrating cncFinder into disease-focused studies may enable the systematic identification of clinically relevant cncRNAs, thereby opening new avenues for biomarker discovery and therapeutic development.

MATERIALS AND METHODS

Benchmark dataset

The positive training dataset, consistent with that used in LncReader, was obtained from cncRNAdb,[17] comprising 1,596 human bifunctional lncRNAs. The positive testing dataset was constructed by integrating the 13 bifunctional lncRNAs from the LncReader testing set with an additional 282 human bifunctional lncRNAs retrieved from codLncDB database,[18] yielding a total of 295 positive testing samples.

The negative dataset was derived from the Ensembl database. Given that current annotations of bifunctional lncRNAs remain incomplete, some transcripts in the negative dataset may potentially exhibit unverified bifunctional activity. To mitigate this label noise, sequences with >80% identity to the positive dataset were removed using CD-HIT.[27] Because the remaining negative dataset is sufficiently large, any undiscovered bifunctional lncRNAs are expected to represent only a very small fraction, and their impact on model training and performance evaluation is therefore negligible. To further reduce redundancy within the negative dataset, CD-HIT was reapplied with an internal sequence identity threshold of 80%. After this process, 295 lncRNAs were randomly selected from the negative dataset to match the size of the positive testing set, to form the negative testing dataset. The remaining non-redundant negative sequences were assigned to the negative training dataset.

Furthermore, to explore the cross-species applicability of cncFinder, 34 bifunctional lncRNAs from *Mus musculus* (mouse) and 6 from *Drosophila melanogaster* (fruit fly) were obtained from cncRNAdb and codLncDB.

The final dataset composition is summarized in Table S2. This dataset configuration not only ensures a fair comparison with LncReader but also provides a robust basis for evaluating the generalizability of cncFinder across independent and cross-species testing sets.

Model architecture

The overall architecture of cncFinder is illustrated in Figure 7, consisting of four components: graph construction, node feature extraction, the GAT layer, and the classification module. First, the graph construction module converted each lncRNA sequence into a directed graph, where nodes represent k-mers and edges are formed based on their sequential adjacency. Subsequently, the node feature extraction module encoded each node using Word2Vec embeddings. The GAT layer then employed an attention mechanism to assign adaptive weights to neighboring nodes, thereby enhancing the learning of structural and functional dependencies within RNA sequences. Finally, the classification module applied an FC layer to predict the bifunctional potential of each transcript.

To prevent overfitting and improve generalizability, cncFinder was trained using 5-fold cross-validation in conjunction with an early stopping strategy. This procedure generated five independent predictive models, and the final prediction was obtained by averaging their outputs.

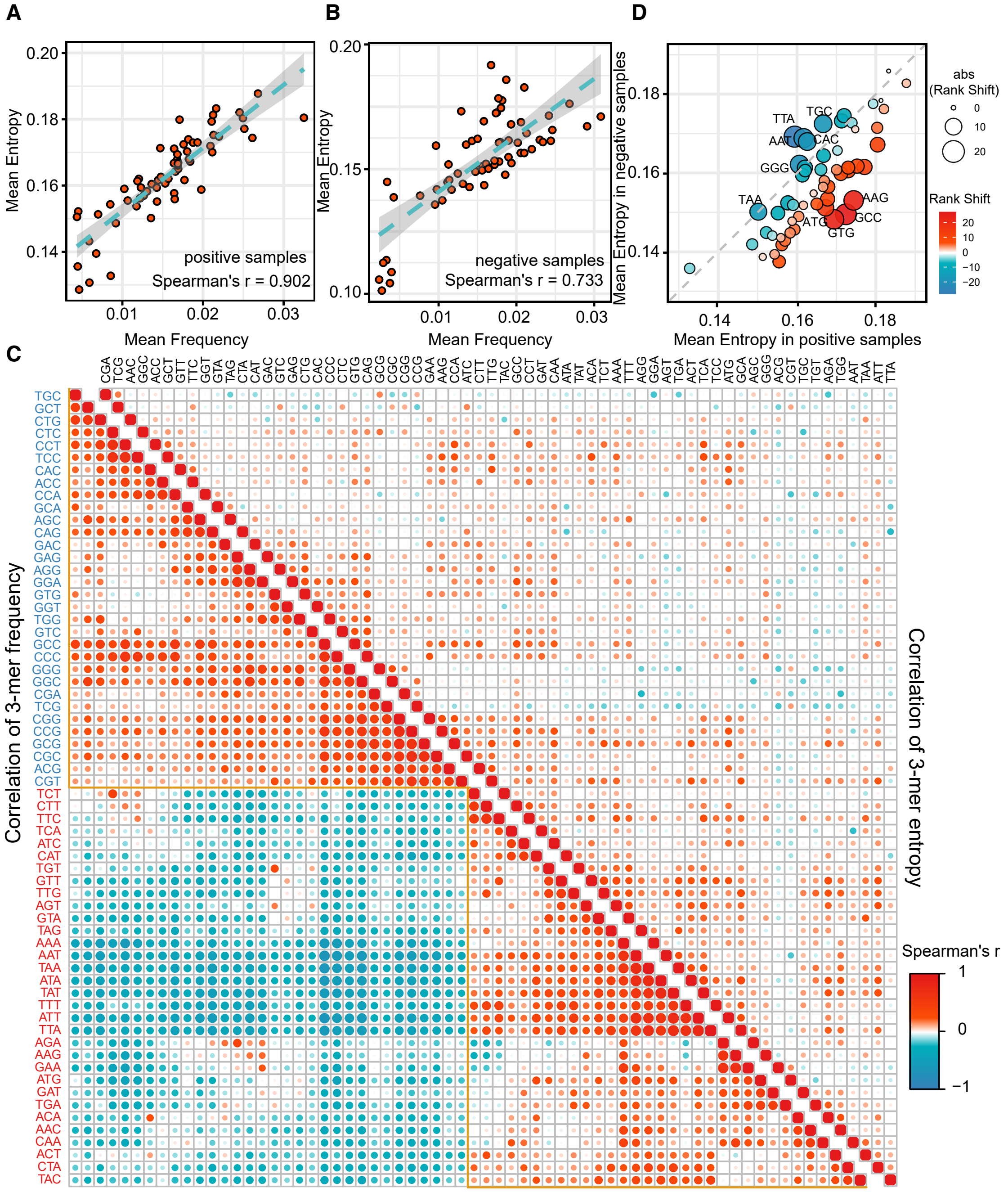

Figure 5. The interpretability analysis of 3-mers in cncFinder

(A and B) Correlation between 3-mer frequency and attention entropy in (A) positive and (B) negative samples. (C) Correlation heatmaps of 3-mer frequency (left) and entropy (right). (D) Rank shift analysis of 3-mer entropy between positive and negative samples.

Graph construction

To capture structural features at the sequence level, the graph construction module utilized a De Bruijn graph to transform lncRNA sequences into directed graphs. As depicted in Figure 7A, a lncRNA sequence of length L is denoted as $N_1N_2N_3N_4 \cdots N_L$, where N corresponds to any nucleotide (A, C, G, or T). Initially, each lncRNA sequence was segmented into k-mer fragments, and edges were established according to overlaps between consecutive k-mer fragments, thereby converting the sequence into a directed graph.

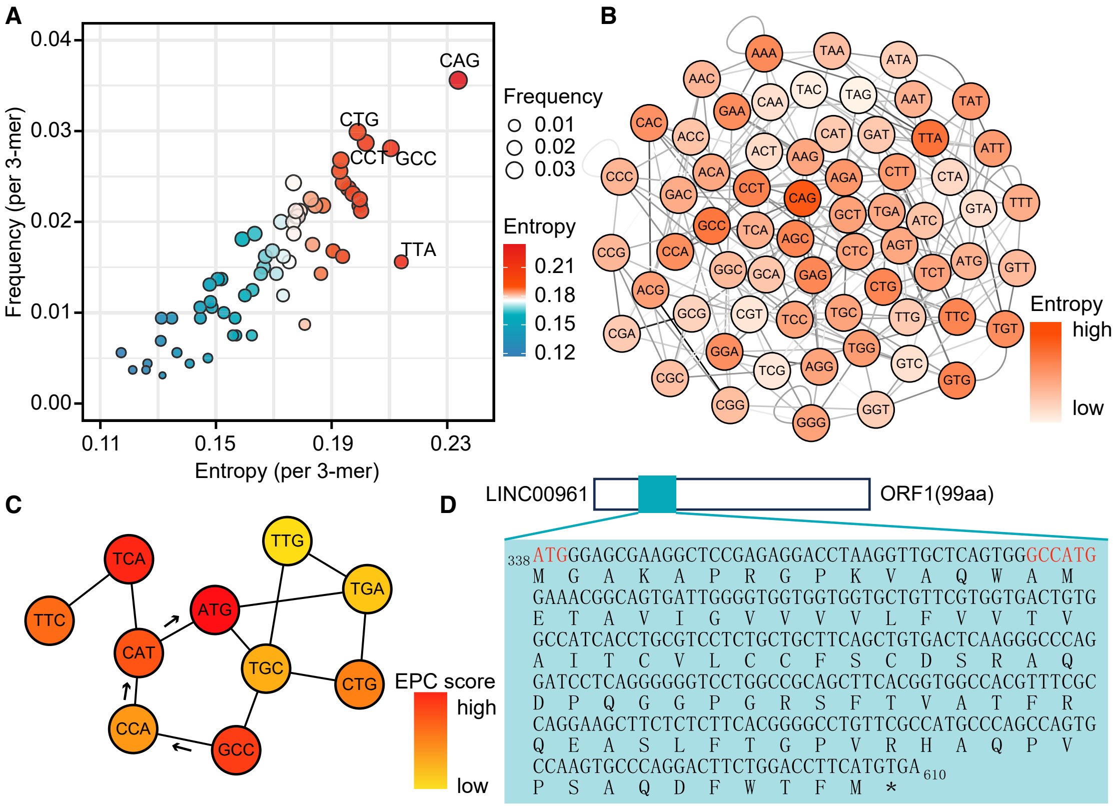

Figure 6. The interpretability analysis of the bifunctional lncRNA LINC00961

(A) Correlation between 3-mer entropy and frequency within LINC00961. (B) 3-mer interaction network constructed based on GAT-derived attention weights. (C) Top 10 ranked 3-mer with the highest network centrality by EPC analysis. (D) The experimentally validated ORF in LINC00961, containing two start codons capable of initiating translation.

Specifically, as illustrated in Figure 7A, for k = 3, the weight of the directed edge from the k-mer $N_1N_2N_3$ to $N_2N_3N_4$ was defined as the frequency with which the 4-mer $N_1N_2N_3N_4$ occurred within the sequence. Subsequently, the edge weights were normalized to attenuate the effect of absolute differences on model training. The normalized formulation was as follows:

$$W_{\text{normalized}} = \frac{W_{ij}}{\sqrt{\sum_{p \in N(i-down)} W_{ip}}\sqrt{\sum_{q \in N(j-up)} W_{qj}}} \quad \text{(Equation 1)}$$

where W_{ij} denotes the original edge weight from node *i* to node *j*. The *N(i-down)* and *N(j-up)* represent the sets of downstream nodes of node *i* and upstream nodes of node j, respectively.

Consequently, the De Bruijn graph can effectively capture local sequence patterns in lncRNAs and maintain the topological relationships among k-mers, thereby furnishing structured input to the GAT model and augmenting its capacity to learn sequence features.

Node feature extraction

Word2Vec is a vector representation learning technique based on a shallow neural network, developed by Mikolov et al. at Google in 2013.[28] Since its introduction, it has emerged as one of the key technologies in distributed representation learning.[29] As depicted in Figure 7B, Word2Vec is utilized to extract node features, thereby enhancing both the structural and semantic representations of lncRNA sequences.

Originally devised for natural language processing, Word2Vec acquires contextual word information through the continuous bag-of-words (CBOW) and skip-gram models. In this study, k-mers were considered analogous to words, and their distributed representations were learned using the CBOW model. Specifically, CBOW predicts the central k-mer by leveraging the contextual information of surrounding k-mers, thereby capturing the local semantic features inherent to lncRNA sequences.

By utilizing the continuous distributed representation of k-mers, cncFinder integrates semantic information into the graph network, enhancing the model's ability to uncover latent relationships among sequences. This representation not only augments the expressiveness of node features but also enhances the capability of the GAT layer to learn structural features of lncRNAs, ultimately boosting the overall predictive performance of the model.

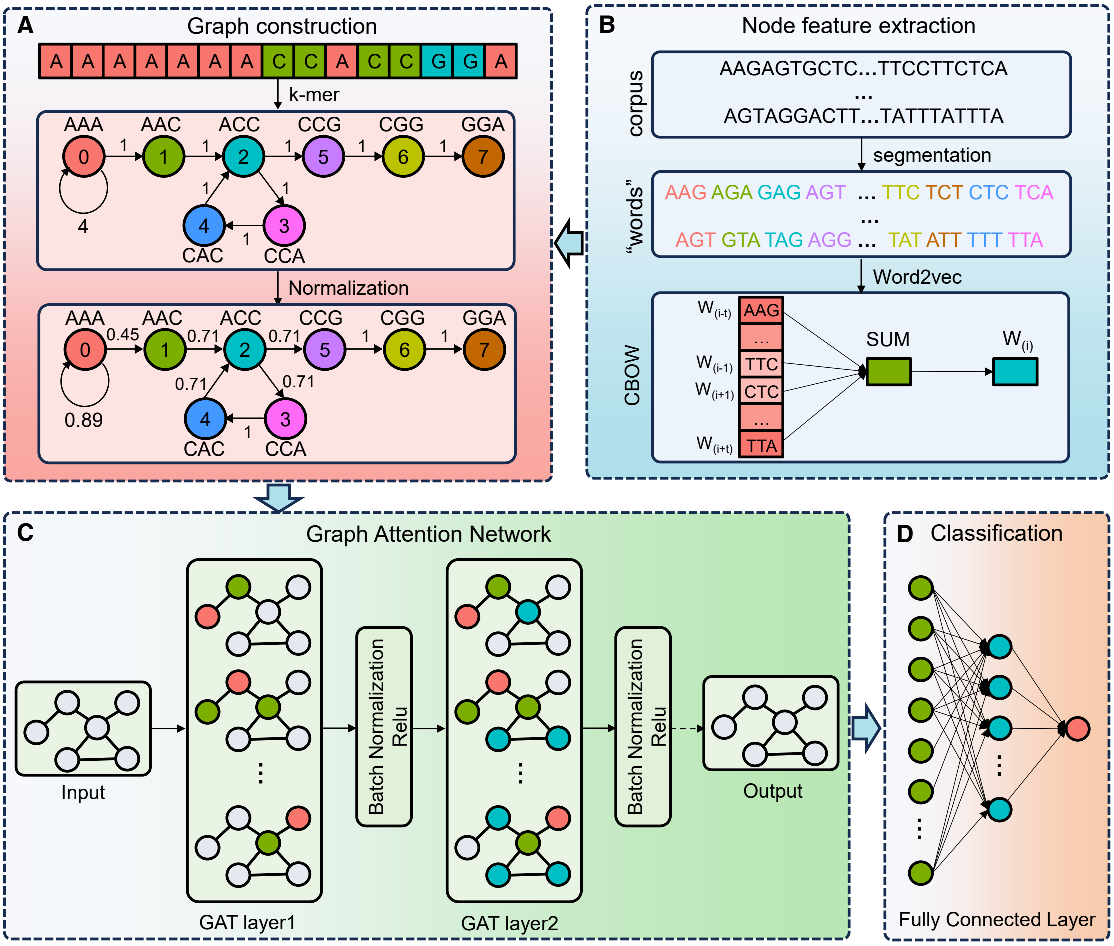

Figure 7. The framework of cncFinder

(A) Graph construction from lncRNA sequences. (B) Node feature extraction using Word2Vec. (C) Feature propagation via GAT layers. (D) Final classification of bifunctional potential via fully connected layers.

Graph attention network

The GAT architecture integrated a multi-head self-attention mechanism to extract high-level node features,[30] as illustrated in Figure 7C. Each attention head was characterized by a distinct set of parameters. The output feature formulation for node i was computed as:

$$h_i' = \|k = 1\ldots,K\left(\alpha_{ii}^k W^k h_i + \sum_{j \in N_i} \alpha_{ij}^k W^k h_j\right) \qquad \text{(Equation 2)}$$

where || denotes the concatenation of outputs from multiple attention mechanisms, K is the number of attention heads, W^k is the weight matrix of the k-th attention mechanism, and α_{ij} denotes the attention coefficient between node i and node j. The attention coefficient α_{ij} was calculated as follows:

$$\alpha_{ij} = \frac{\exp\left(\mathrm{LeakyReLU}\left(a^{\mathrm{T}}\left[Wh_i \| Wh_j\right]\right)\right)}{\sum_{k \in N_i} \exp\left(\mathrm{LeakyReLU}\left(a^{\mathrm{T}}\left[Wh_i \| Wh_k\right]\right)\right)} \qquad \text{(Equation 3)}$$

where a^{T} denotes a learnable weight vector, and ReLU is a nonlinear activation function.

To quantify the contextual variability of each 3-mer node, we further computed the attention entropy based on the edge attention weights derived from the GAT layer. The attention entropy H_i of node i was defined using the Shannon entropy formula:

$$H_i = -\sum_{j \in N(i)} \alpha_{ij} \log_2\left(\alpha_{ij}\right) \qquad \text{(Equation 4)}$$

where $N(i)$ represents the set of all neighbors of node i. A higher entropy value reflects a more diverse distribution of attention across neighboring nodes, indicating that the node integrates information from a broader contextual environment. Conversely, a lower entropy suggests that attention is more concentrated on a limited number of neighbors.

Finally, as shown in Figure 7D, the high-level features extracted via the GAT layer were fed into multiple FC layers to execute the classification task.

Performance evaluation and hyperparameter tuning

To comprehensively evaluate the performance of cncFinder, we employed multiple metrics, including ACC, SN, SP, and MCC.[31] These evaluation metrics were calculated as follows:

$$ACC = \frac{TP + TN}{TP + FP + TN + FN} \quad \text{(Equation 5)}$$

$$SN = \frac{TP}{TP + FN} \quad \text{(Equation 6)}$$

$$SP = \frac{TN}{TN + FP} \quad \text{(Equation 7)}$$

$$MCC = \frac{TP \times TN - FP \times FN}{\sqrt{(TP + FP)(TP + FN)(TN + FP)(TN + FN)}} \quad \text{(Equation 8)}$$

where TP, TN, FP, and FN represent the number of true positives, true negatives, false positives, and false negatives, respectively. Additionally, AUC was employed to assess the model's discriminative ability.

To further evaluate the model stability, we conducted a sensitivity analysis on a set of key hyperparameters, including k-mer size, Word2Vec embedding dimension, number of hidden layer neurons, and number of attention heads. The ranges of these hyperparameter perturbations are summarized in Table S3.

cncFinder was trained using the Adam optimizer with a learning rate of 1×10^{-4}, batch size of 64, and 200 epochs. All experiments were conducted on an NVIDIA RTX A100 GPU with 80 GB memory under the PyTorch 1.1.3 deep learning framework.

DATA AVAILABILITY

The datasets and source code are freely available at https://github.com/TangQiang0701/cncFinder.

ACKNOWLEDGMENTS

This work was supported by National Natural Science Foundation of China (no. 62302066), Natural Science Foundation of Sichuan Province (no. 2024NSFSC1295), Guangdong Basic and Applied Basic Research Foundation (no. 2021A1515110653), Chengdu Medical College Technology Program (no. KYPY25-03), and Chengdu Medical College Excellent-talent Program (no. 2024kjTzn01).

AUTHOR CONTRIBUTIONS

Q.T., methodology, data curation, formal analysis, writing—original draft, and funding acquisition. Y.Y., resources and validation. M.S., resources and visualization. L.Z., formal analysis and visualization. X.J., supervision, funding acquisition, and writing—review & editing. J.K., conceptualization, funding acquisition, writing—review & editing, and project administration.

DECLARATION OF INTERESTS

The authors declare no competing interests.

DECLARATION OF GENERATIVE AI AND AI-ASSISTED TECHNOLOGIES IN THE WRITING PROCESS

During the preparation of this work, the authors used ChatGPT 4.0 to improve language and readability. After using this tool, the authors reviewed and edited the content as needed and take full responsibility for the content of the publication.

SUPPLEMENTAL INFORMATION

Supplemental information can be found online at https://doi.org/10.1016/j.omtn.2025.102812.

REFERENCES

1. Hube, F., and Francastel, C. (2018). Coding and Non-coding RNAs, the Frontier Has Never Been So Blurred. Front. Genet. *9*, 140.
2. Kumari, P., and Sampath, K. (2015). cncRNAs: Bi-functional RNAs with protein coding and non-coding functions. Semin. Cell Dev. Biol. *47–48*, 40–51.
3. Sampath, K., and Ephrussi, A. (2016). CncRNAs: RNAs with both coding and non-coding roles in development. Development *143*, 1234–1241.
4. Chen, J., Brunner, A.D., Cogan, J.Z., Nuñez, J.K., Fields, A.P., Adamson, B., Itzhak, D.N., Li, J.Y., Mann, M., Leonetti, M.D., and Weissman, J.S. (2020). Pervasive functional translation of noncanonical human open reading frames. Science *367*, 1140–1146.
5. Huang, J.Z., Chen, M., Chen, D., Gao, X.C., Zhu, S., Huang, H., Hu, M., Zhu, H., and Yan, G.R. (2017). A Peptide Encoded by a Putative lncRNA HOXB-AS3 Suppresses Colon Cancer Growth. Mol. Cell *68*, 171–184.e6.
6. Cai, B., Li, Z., Ma, M., Wang, Z., Han, P., Abdalla, B.A., Nie, Q., and Zhang, X. (2017). LncRNA-Six1 Encodes a Micropeptide to Activate Six1 in Cis and Is Involved in Cell Proliferation and Muscle Growth. Front. Physiol. *8*, 230.
7. Matsumoto, A., Pasut, A., Matsumoto, M., Yamashita, R., Fung, J., Monteleone, E., Saghatelian, A., Nakayama, K.I., Clohessy, J.G., and Pandolfi, P.P. (2017). mTORC1 and muscle regeneration are regulated by the LINC00961-encoded SPAR polypeptide. Nature *541*, 228–232.
8. Lu, S., Zhang, J., Lian, X., Sun, L., Meng, K., Chen, Y., Sun, Z., Yin, X., Li, Y., Zhao, J., et al. (2019). A hidden human proteome encoded by 'non-coding' genes. Nucleic Acids Res. *47*, 8111–8125.
9. Slavoff, S.A., Mitchell, A.J., Schwaid, A.G., Cabili, M.N., Ma, J., Levin, J.Z., Karger, A.D., Budnik, B.A., Rinn, J.L., and Saghatelian, A. (2013). Peptidomic discovery of short open reading frame-encoded peptides in human cells. Nat. Chem. Biol. *9*, 59–64.
10. Calviello, L., Mukherjee, N., Wyler, E., Zauber, H., Hirsekorn, A., Selbach, M., Landthaler, M., Obermayer, B., and Ohler, U. (2016). Detecting actively translated open reading frames in ribosome profiling data. Nat. Methods *13*, 165–170.
11. Calviello, L., Hirsekorn, A., and Ohler, U. (2020). Quantification of translation uncovers the functions of the alternative transcriptome. Nat. Struct. Mol. Biol. *27*, 717–725.
12. Makarewich, C.A., and Olson, E.N. (2017). Mining for Micropeptides. Trends Cell Biol. *27*, 685–696.
13. Zhang, Z.Y., Zhang, Z., Ye, X., Sakurai, T., and Lin, H. (2024). A BERT-based model for the prediction of lncRNA subcellular localization in Homo sapiens. Int. J. Biol. Macromol. *265*, 130659.
14. Mohapatra, M., Sahu, C., and Mohapatra, S. (2025). Trends of Artificial Intelligence (AI) Use in Drug Targets, Discovery and Development: Current Status and Future Perspectives. Curr. Drug Targets *26*, 221–242.
15. Tang, Q., and Chen, W. (2025). DeepB(3)P: A transformer-based model for identifying blood-brain barrier penetrating peptides with data augmentation using feedback GAN. J. Adv. Res. *73*, 459–468.
16. Kang, J., Yu, S., Lu, S., Xu, G., Zhu, J., Yan, N., Luo, D., Xu, K., Zhang, Z., and Huang, J. (2020). Use of a 6-miRNA panel to distinguish lymphoma from reactive lymphoid hyperplasia. Signal Transduct. Target. Ther. *5*, 2.
17. Huang, Y., Wang, J., Zhao, Y., Wang, H., Liu, T., Li, Y., Cui, T., Li, W., Feng, Y., Luo, J., et al. (2021). cncRNAdb: a manually curated resource of experimentally supported

RNAs with both protein-coding and noncoding function. Nucleic Acids Res. *49*, D65–D70.

18. Liu, T., Qiao, H., Wang, Z., Yang, X., Pan, X., Yang, Y., Ye, X., Sakurai, T., Lin, H., and Zhang, Y. (2024). CodLncScape Provides a Self-Enriching Framework for the Systematic Collection and Exploration of Coding LncRNAs. Adv. Sci. *11*, e2400009.
19. Liu, T., Zou, B., He, M., Hu, Y., Dou, Y., Cui, T., Tan, P., Li, S., Rao, S., Huang, Y., et al. (2023). LncReader: identification of dual functional long noncoding RNAs using a multi-head self-attention mechanism. Brief. Bioinform. *24*, bbac579.
20. Kang, Y.J., Yang, D.C., Kong, L., Hou, M., Meng, Y.Q., Wei, L., and Gao, G. (2017). CPC2: a fast and accurate coding potential calculator based on sequence intrinsic features. Nucleic Acids Res. *45*, W12–W16.
21. Li, A., Zhang, J., and Zhou, Z. (2014). PLEK: a tool for predicting long non-coding RNAs and messenger RNAs based on an improved k-mer scheme. BMC Bioinf. *15*, 311.
22. Cherrak, Y., Salazar, M.A., Näpflin, N., Malfertheiner, L., Herzog, M.K.M., Schubert, C., von Mering, C., and Hardt, W.D. (2024). Non-canonical start codons confer context-dependent advantages in carbohydrate utilization for commensal E. coli in the murine gut. Nat. Microbiol. *9*, 2696–2709.
23. Xie, J., Zhuang, Z., Gou, S., Zhang, Q., Wang, X., Lan, T., Lian, M., Li, N., Liang, Y., Ouyang, Z., et al. (2023). Precise genome editing of the Kozak sequence enables bidirectional and quantitative modulation of protein translation to anticipated levels without affecting transcription. Nucleic Acids Res. *51*, 10075–10093.
24. Sun, L., Luo, H., Bu, D., Zhao, G., Yu, K., Zhang, C., Liu, Y., Chen, R., and Zhao, Y. (2013). Utilizing sequence intrinsic composition to classify protein-coding and long non-coding transcripts. Nucleic Acids Res. *41*, e166.
25. Rood, J.E., Wynne, S., Robson, L., Hupalowska, A., Randell, J., Teichmann, S.A., and Regev, A. (2025). The Human Cell Atlas from a cell census to a unified foundation model. Nature *637*, 1065–1071.
26. Liu, T., Qiao, H., Ren, L., Ye, X., Zou, Q., and Zhang, Y. (2025). Deciphering cancer therapy resistance via patient-level single-cell transcriptomics with CellResDB. Commun. Biol. *8*, 1049.
27. Li, W., and Godzik, A. (2006). Cd-hit: a fast program for clustering and comparing large sets of protein or nucleotide sequences. Bioinformatics *22*, 1658–1659.
28. Tomas Mikolov, K.C., Corrado, G., and Jeffrey, D. (2013). Efficient Estimation of Word Representations in Vector Space. Preprint at arXiv. https://doi.org/10.48550/arXiv.1301.3781.
29. Wei, Z., Shen, Y., Tang, X., Wen, J., Song, Y., Wei, M., Cheng, J., and Zhu, X. (2025). AVPpred-BWR: antiviral peptides prediction via biological words representation. Bioinformatics *41*, btaf126.
30. Abdelkader, G.A., and Kim, J.D. (2024). Advances in Protein-Ligand Binding Affinity Prediction via Deep Learning: A Comprehensive Study of Datasets, Data Preprocessing Techniques, and Model Architectures. Curr. Drug Targets *25*, 1041–1065.
31. Liu, T., Huang, J., Luo, D., Ren, L., Ning, L., Huang, J., Lin, H., and Zhang, Y. (2024). Cm-siRPred: Predicting chemically modified siRNA efficiency based on multi-view learning strategy. Int. J. Biol. Macromol. *264*, 130638.

OMTN, Volume *37*

Supplemental information

cncFinder: A graph-attention-network-based interpretable learning model to identify bifunctional long non-coding RNAs

Qiang Tang, Yang Yu, Min Shen, Lin Zhang, Xu Jia, and Juanjuan Kang

Supplemental information

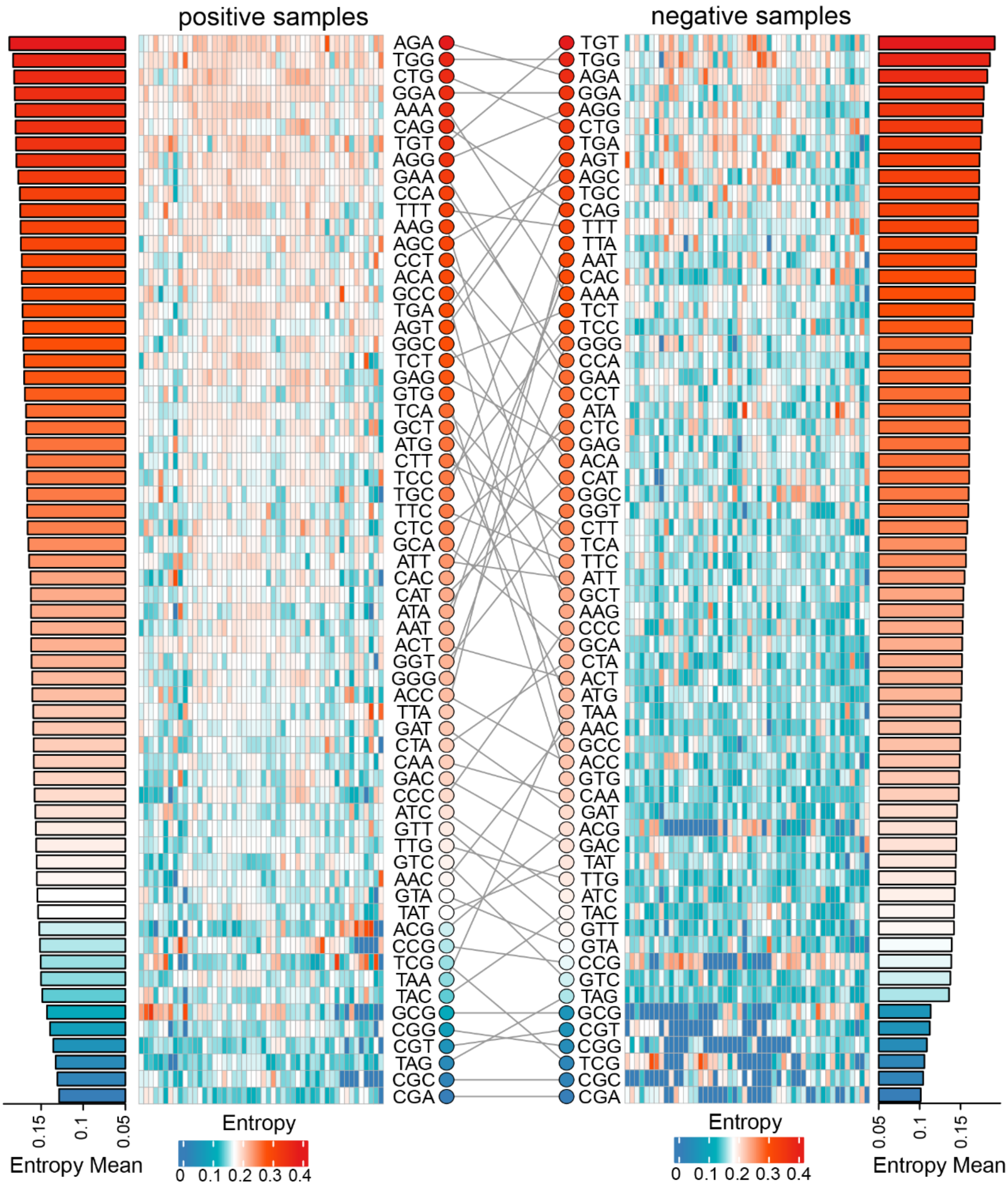

Figure S1 Comparison of 3-mer entropy distributions between positive and negative samples. Left: Bar plot showing the average 3-mer attention entropy across 1,596 experimentally validated bifunctional lncRNAs. Heatmap displays the 3-mer entropy patterns for a randomly selected subset of 50 bifunctional lncRNAs. Right: Bar plot showing the average 3-mer entropy across 1,596 predicted non-bifunctional lncRNAs with the lowest prediction probabilities. Heatmap displays the 3-mer entropy for a randomly selected subset of 50 lncRNAs from this group.

Table S1 Ablation Study.

	Testing dataset					Cross-species dataset
Methods	SN	SP	ACC	MCC	AUC	ACC (No. correctly predicted)
Doc2Vec	0.593	0.939	0.766	0.567	0.869	0.425(17)
fastText	0.793	0.864	0.829	0.659	0.873	0.750(30)
TF-IDF	0.885	0.563	0.724	0.473	0.854	0.825(33)
w/o GAT	0.509	0.966	0.737	0.534	0.892	0.350(14)
GCN	0.505	0.953	0.729	0.512	0.868	0.275(11)
cncFinder	0.851	0.861	0.856	0.712	0.883	0.850(34)

Table S2 Comprehensive overview of dataset employed for cncFinder training and testing.

Datasets	Species	Positive sequences	Negative sequences
Training	Human	1,596	28,901
Testing	Human	295	295
Cross species	Mouse	34	-
	Fruit fly	6	-

Table S3 The hyperparameter perturbations for cncFinder.

Hyperparameters	Range
k-mer size	3, 4, 5, 6
Word2Vec embedding vector dimension	64, 128, 256, 512
number of hidden neurons	64, 128, 256, 512
number of attention heads	3, 4, 5
